# Prediction of mortality in patients with colorectal perforation based on routinely available parameters: a retrospective study

**DOI:** 10.1186/s13017-015-0020-y

**Published:** 2015-06-25

**Authors:** Takehito Yamamoto, Ryosuke Kita, Hideyuki Masui, Hiromitsu Kinoshita, Yusuke Sakamoto, Kazuyuki Okada, Junji Komori, Akira Miki, Kenji Uryuhara, Hiroyuki Kobayashi, Hiroki Hashida, Satoshi Kaihara, Ryo Hosotani

**Affiliations:** Department of Surgery, Kitano Hospital, The Tazuke Kofukai Medical Research Institute, 2-4-20 Ogimachi, Kita-ku, Osaka, 530-8480 Japan; Kobe City Medical Center General Hospital, 2-1-1 Minatojima-Minamimachi, Chuoku, Kobe 650-0047 Japan

**Keywords:** Colorectal perforation, Mortality marker, Prognostic factor

## Abstract

**Introduction:**

Even after surgery and intensive postoperative management, the mortality rate associated with colorectal perforation is high. Identification of mortality markers using routinely available preoperative parameters is important.

**Methods:**

We enrolled consecutive patients with colorectal perforation who underwent operations from January 2010 to January 2015. We divided them into a mortality and survivor group and compared clinical characteristics between the two groups. Additionally, we compared the mortality rate between different etiologies: malignant versus benign and diverticular versus nondiverticular. We used the *χ*^2^ and Mann–Whitney U tests and a logistic regression model to identify factors associated with mortality.

**Results:**

We enrolled 108 patients, and 52 (48 %) were male. The mean age at surgery was 71 ± 13 years. The postoperative mortality rate was 12 % (13 patients). Multivariate logistic regression analysis showed that a high patient age (odds ratio [OR], 1.09; 95 % confidence interval [CI], 1.020–1.181) and low preoperative systolic blood pressure (OR, 0.98; 95 % CI, 0.953–0.999) were independent risk factors for mortality in patients with colorectal perforation. In the subgroup analysis, there was no significant difference between the malignant and benign group (11.8 % vs. 23.9 %, respectively; *p* = 0.970), while the diverticular group had a significantly lower mortality rate than the nondiverticular group (2.6 % vs. 17.1 %, respectively; *p* = 0.027).

**Conclusions:**

Older patients and patients with low preoperative blood pressure had a high risk of mortality associated with colorectal perforation. For such patients, operations and postoperative management should be performed carefully.

## Introduction

Colorectal perforation causes widespread dissemination of bacteria throughout the intra-abdominal space and easily leads to panperitonitis and septic shock. Septic shock is responsible for disseminated intravascular coagulation and organ failure. Therefore, the mortality rate associated with colorectal perforation is considered to be high. The Complicated Intra-Abdominal infections Worldwide Observational (CIAOW) study, a large multicenter observational study that included 1898 patients undergoing surgery or interventional drainage for complicated intra-abdominal infections performed by Sartelli et al. [1], indicated that colonic nondiverticular perforation was a source of infection that was significantly correlated with patient mortality. Many other studies also analyzed the mortality of colorectal perforation, and the reported mortality rate ranged from 6 to 33 % [2–10]. Immediate surgical management of colorectal perforation is necessary, and preoperative knowledge of the severity of colorectal perforation and risk factors for mortality is also important. Patients with severe peritonitis should undergo preoperative preparations for high-quality postoperative intensive care. Additionally, adequate information about the likelihood of mortality should be provided to the patient and his or her family before the operation.

A number of studies have reported several risk factors for mortality associated with colorectal perforation, such as age, sex, the serum protein level, and the serum creatinine level [2, 5, 7, 8]. However, most such studies involved small samples or were performed many years ago. For example, Alvarez et al. [2] enrolled 114 patients from 1986 to 2005, and Kriwanek et al. [7] enrolled 112 patients from 1979 to 1992. These study periods were too long and included many old cases. On the other hand, a recent study by Shimazaki et al. [8] was performed from 1998 to 2011, but they enrolled just 42 patients. We consider that postoperative intensive care techniques are progressing year by year, and a study enrolling many patients within a short period is necessary in this field. Our institution has a large emergency care unit and serves as the core emergency medicine facility in the region; thus, many patients with colorectal perforation present at our institution every year. Therefore, we analyzed mortality markers in consecutive patients with colorectal perforation who underwent operations in our institution.

## Patients and methods

### Data collection

We analyzed consecutive patients with colorectal perforation who underwent emergent surgery from January 2010 to January 2015 at a single center. All patients underwent surgery within 24 h of diagnosis. The primary outcome was mortality after surgery. The patients were divided into a mortality group and a survivor group, and we analyzed the factors associated with mortality. Mortality was defined as death of colorectal perforation within 2 months after surgery. The patients’ clinical characteristics were reviewed, including age, sex, body mass index, comorbidities, preoperative laboratory data, etiology, and site of perforation.

Additionally, we divided the patients into two groups according to etiology (malignant versus benign and diverticular versus nondiverticular) and compared the mortality rates between these groups. Furthermore, as a subgroup analysis, we analyzed risk factors associated with mortality in the nondiverticular group alone.

The preoperative white blood cell counts were dichotomized into ≥4000/μL, <4000 to ≤12,000/μL, and >12,000/μL, and the preoperative body temperature was dichotomized into ≤36 °C, 36 °C to ≤38 °C, and >38 °C, which reflect the criteria for systemic inflammatory response syndrome criteria [11]. Other variables were evaluated as continuous variables.

### Diagnosis

Colorectal perforation was diagnosed by two or more surgeons and radiologists according to the following criteria: a) the presence of symptoms indicating panperitonitis, such as severe abdominal pain and nausea; b) the presence of signs of peritoneal irritation such as muscular defense and rebound tenderness, indicating panperitonitis; and c) the presence of free air on preoperative computed tomography. Abdominal radiography findings were not useful in diagnosing colorectal perforation and were not included in the diagnostic criteria. All perforations were diagnosed preoperatively and confirmed intraoperatively.

### Statistical analyses

Continuous variables are presented as mean ± standard deviation or median (range), and categorical variables are expressed as number and percentage. We used the *χ*^2^ and Mann–Whitney U tests and a logistic regression model to assess the associations between outcomes and clinical characteristics. The factors with a significant relationship in the univariate analyses were subsequently used in the multivariate regression models. The effect of a factor was presented as the odds ratio (OR) and its 95 % confidence interval (CI). All statistical analyses were conducted by one physician (T.Y.) using JMP 10 (SAS Institute Inc., Cary, NC, USA). A p value of <0.05 was considered statistically significant.

The protocol for this study was approved by our hospital’s institutional review board. Informed consent was waived because of the historical cohort nature of the study.

## Results

**Fig. 1:**
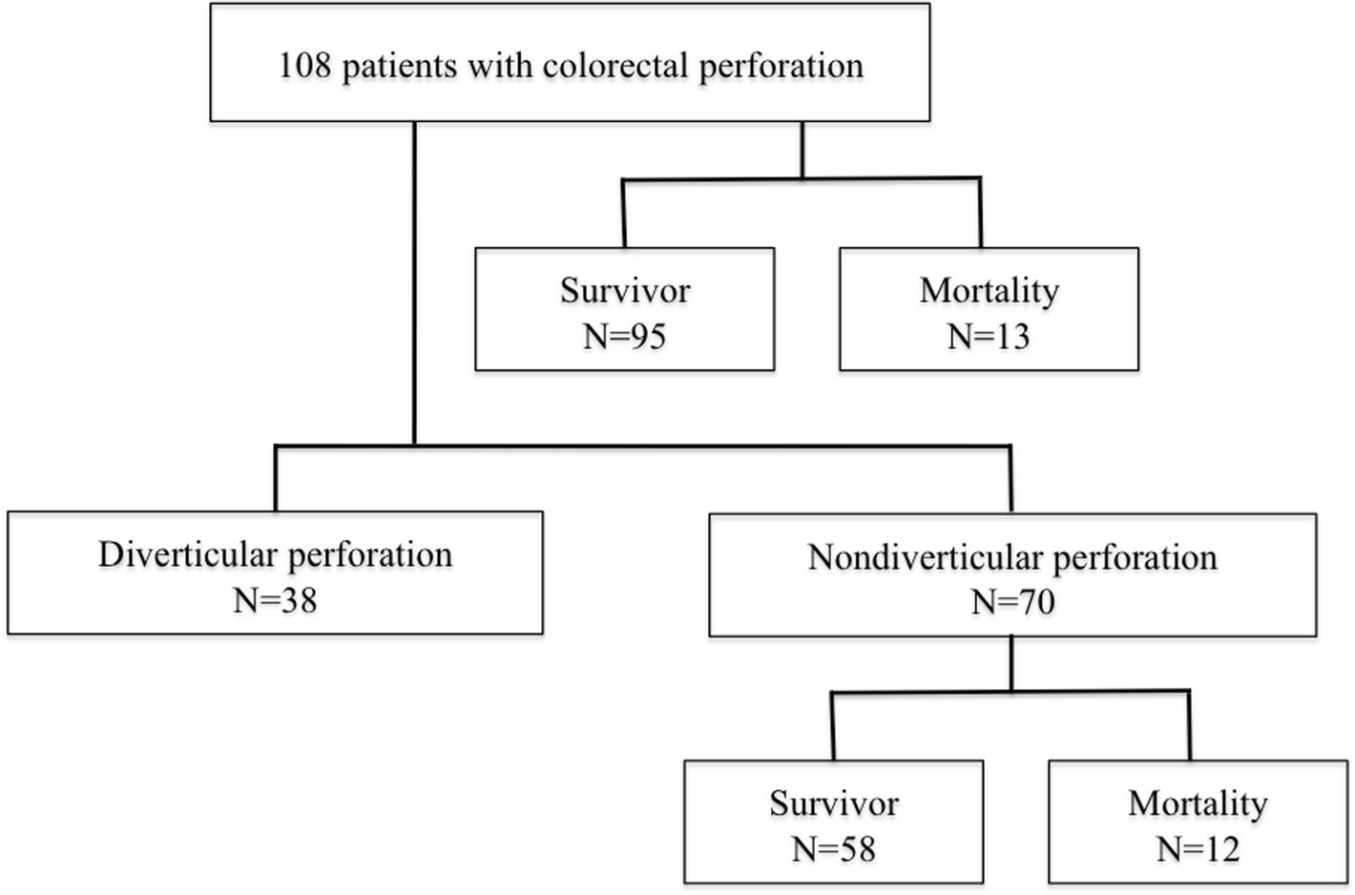
Patient selection

Patient selection is shown in Fig. 1. We enrolled 108 patients, whose clinical characteristics are presented in Table 1. Their mean age at surgery was 71 ± 13 years, and 52 (48 %) were male. The etiology of the perforation was a diverticulum (*n* = 38), cancer (*n* = 17), fecal impaction (*n* = 17), iatrogenic (*n* = 12), inflammatory disease (*n* = 5), trauma (*n* = 2), rectal prolapse (*n* = 1), and idiopathic (*n* = 16). All perforative fecal impactions were caused by colorectal obstruction and obstructive colitis by retention of feces and chronic constipation, which were confirmed intraoperatively. Iatrogenic perforations included perforations due to colonoscopy and radiographic contrast enemas, and inflammatory diseases included cytomegalovirus-induced colitis, ulcerative colitis, Behçet’s disease, and colitis caused by radiation therapy for rectal cancer.

**Table 1:** Clinical characteristics of patients

Variables	
Age (years)	71 ± 13
Male	52 (48.1)
Body mass index (kg/m^2^)	21.5 ± 4.6
Chemotherapy	8 (7.4)
Steroid use	25 (23.1)
Diabetes mellitus	7 (6.5)
Body temperature >38.0 °C or <36.0 °C	34 (31.4)
Systolic blood pressure (mmHg)	127 (50–188)
Heart rate (/min)	98 (54–144)
White blood cells >12,000 or <4000 (/μL)	50 (46.2)
C-reactive protein (mg/dl)	11.7 ± 10.7
Albumin (mg/dl)	2.8 ± 0.8
Creatinine (mg/dl)	1.2 ± 1.2
Perforation etiology	
Diverticulum	38 (35.2)
Cancer	17 (15.7)
Fecal impaction	17 (15.7)
Iatrogenic	12 (11.1)
Inflammatory disease	5 (4.6)
Trauma	2 (1.9)
Rectal prolapse	1 (0.9)
Idiopathic	16 (14.8)
Perforation site	
Cecum	8 (7.4)
Ascending colon	4 (3.7)
Transverse colon	7 (6.5)
Descending colon	6 (5.6)
Sigmoid colon	70 (64.8)
Rectum	13 (12.0)
Stoma creation	84 (77.8)
Length of operation (min)	179 ± 57
Blood loss (g)	219 (0–3112)
Hospital stay (d)	19 (4–185)

Data are presented as mean ± standard deviation, median (range), or n (%)

**Fig. 2:**
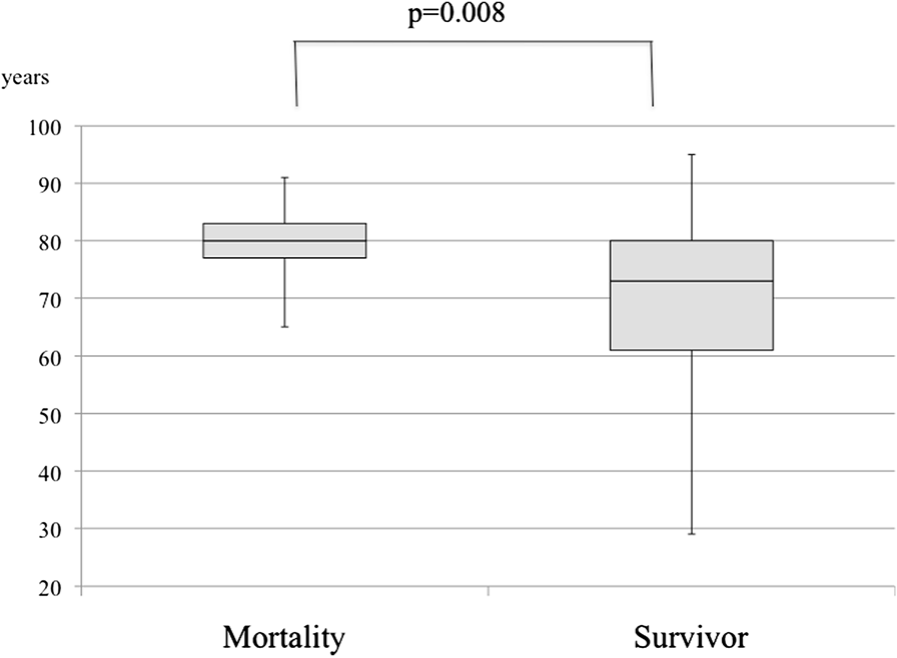
Comparison of age between mortality group and survivor group. Patients were significantly older in the mortality group than in the survivor group (79 ± 8 vs. 70 ± 13 years, respectively; *p* = 0.008)

**Fig. 3:**
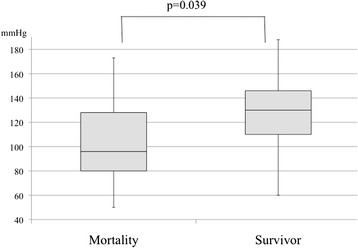
Comparison of preoperative systolic blood pressure between mortality group and survivor group. Preoperative systolic blood pressure was significantly lower in the mortality group than in the survivor group (96 vs. 130 mmHg, respectively; *p* = 0.039)

The postoperative mortality rate was 12 % (13 patients). The clinical characteristics between the survivor group and mortality group are compared in Table 2. The patients were significantly older in the mortality group than in the survivor group (79 ± 8 vs. 70 ± 13 years, respectively; *p* = 0.009). The mean preoperative systolic blood pressure was significantly lower in the mortality group than in the survivor group (96 vs. 130 mmHg, respectively; *p* = 0.004). The mean preoperative serum creatinine level was significantly higher in the morality group than in the survivor group (2.0 ± 1.4 vs. 1.1 ± 1.2 mg/dl, respectively; *p* = 0.004). Logistic regression analysis using the potential risk factors for mortality determined by univariate analysis (patient age, preoperative systolic blood pressure, and preoperative serum creatinine level) showed that patient age (OR, 1.09; 95 % CI, 1.020–1.181) and preoperative systolic blood pressure (OR, 0.98; 95 % CI, 0.953–0.999) were independent risk factors for mortality in patients with colorectal perforation (Table 3). Figures 2 and 3 compare these two factors between the survivor group and mortality group.

**Table 2:** Comparison of clinical characteristics between the survivor and mortality groups

Variables	Survivor	Mortality	*p* value
	*n* = 95	*n* = 13	
Age (years)	70 ± 13	79 ± 8	0.009^*^
Male	48	4	0.181
Body mass index (kg/m^2^)	21.8 ± 4.7	19.5 ± 3.3	0.080
Chemotherapy	6	2	0.242
Steroid use	20	5	0.163
Diabetes mellitus	5	2	0.169
Body temperature >38.0 °C or <36.0 °C	30	4	
Systolic blood pressure (mmHg)	130 (60–188)	96 (50–173)	0.004^*^
Heart rate (/min)	96 (54–144)	110 (75–133)	0.074
White blood cells >12,000 or <4000 (/μL)	45	5	0.546
C-reactive protein (mg/dl)	12.4 ± 11.0	6.8 ± 6.8	0.171
Albumin (mg/dl)	2.9 ± 0.8	2.5 ± 0.7	0.058
Creatinine (mg/dl)	1.1 ± 1.2	2.0 ± 1.4	0.004^*^
Perforation etiology			0.275
Diverticulum	37	1	
Cancer	15	2	
Fecal impaction	13	4	
Iatrogenic	11	1	
Inflammatory disease	4	1	
Trauma	2	0	
Rectal prolapse	1	0	
Idiopathic	12	4	
Perforation site			0.055
Cecum	7	1	
Ascending colon	3	1	
Transverse colon	7	0	
Descending colon	3	3	
Sigmoid colon	62	8	
Rectum	13	0	
Stoma creation	72	12	0.179
Length of operation (min)	177 ± 55	187 ± 73	0.581
Blood loss (g)	200 (0–3112)	347 (0–1000)	0.248

Data are presented as mean ± standard deviation, median (range), or n

^*^ *p* < 0.05

**Table 3:** Multivariate logistic regression analysis of risk factors for mortality of colorectal perforation

Variables	Odds ratio	95 % Confidence interval	*p* value
Age	1.09	1.020–1.181	0.008^*^
Systolic blood pressure	0.98	0.953–0.999	0.039^*^
Creatinine	1.43	0.918–2.206	0.107

^*^ *p* < 0.05

**Fig. 4:**
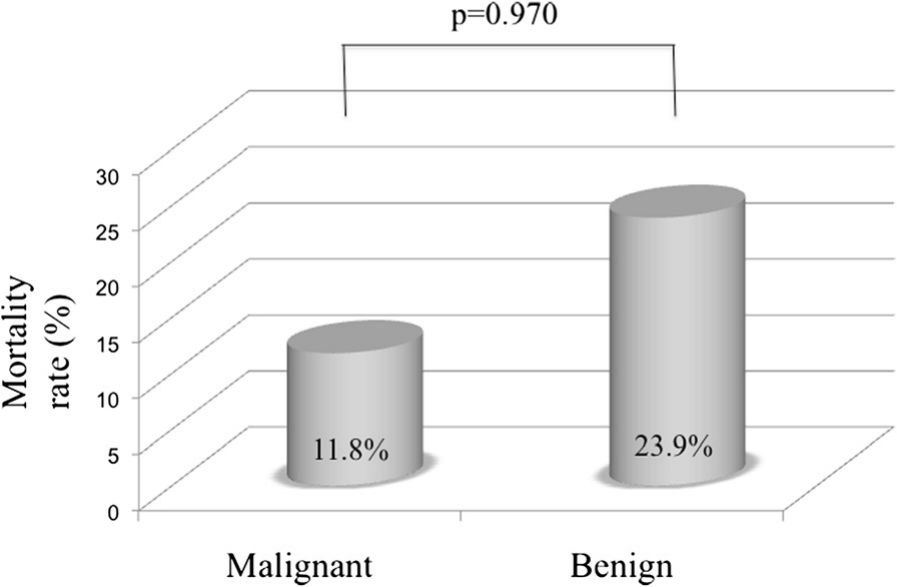
Comparison of mortality rate between malignant group and benign group. There was no significant difference in the mortality rate between the two groups (11.8 % vs. 23.9 %, respectively; *p* = 0.970)

**Fig. 5:**
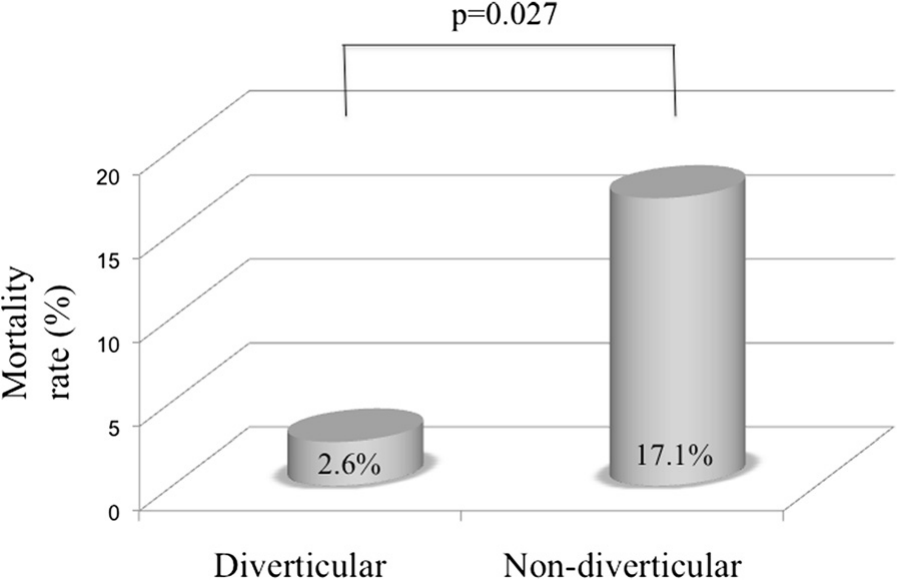
Comparison of mortality rate between diverticular group and nondiverticular group. The mortality rate of the diverticular group was significantly lower than that of the nondiverticular group (2.6 % vs. 17.1 %, respectively; *p* = 0.027)

The comparison of the mortality rate between the malignant and benign groups is shown in Fig. 4. There was no significant difference in the mortality rate between the two groups (11.8 % vs. 23.9 %, respectively; *p* = 0.970). The comparison of the mortality rate between the diverticular and nondiverticular groups is shown in Fig. 5. The mortality rate in the diverticular group was significantly lower than that in the nondiverticular group (2.6 % vs. 17.1 %, respectively; *p* = 0.027).

The results of subgroup analysis in the nondiverticular group (*n* = 70) are shown in Tables 4 and 5. In the univariate analysis, older age, lower systolic blood pressure, higher heart rate, and higher serum creatinine level were significantly associated with mortality, and the multivariate analysis showed that age alone was a significant mortality marker (OR, 1.09; 95 % CI, 1.013–1.182).

**Table 4:** Comparison of clinical characteristics between the survivor and mortality groups in the nondiverticular group

Variables	Survivor	Mortality	*p* value
	*n* = 58	*n* = 12	
Age (years)	72 ± 12	81 ± 7	0.008^*^
Male	25	4	0.532
Body mass index (kg/m^2^)	20.4 ± 4.0	19.4 ± 3.4	0.428
Chemotherapy	4	2	0.271
Steroid use	10	4	0.228
Diabetes mellitus	4	2	0.271
Body temperature >38.0 °C or <36.0 °C	14	3	0.950
Systolic blood pressure (mmHg)	131 (60–188)	95 (50–173)	0.004^*^
Heart rate (/min)	93 (54–144)	110 (75–133)	0.031^*^
White blood cells >12,000 or <4000 (/μL)	29	4	0.288
C-reactive protein (mg/dl)	10.8 ± 11.7	6.3 ± 6.9	0.624
Albumin (mg/dl)	2.8 ± 0.8	2.5 ± 0.7	0.142
Creatinine (mg/dl)	1.1 ± 1.1	1.7 ± 0.9	0.010^*^
Perforation site			0.113
Cecum	4	1	
Ascending colon	2	1	
Transverse colon	6	0	
Descending colon	3	3	
Sigmoid colon	31	7	
Rectum	12	0	
Stoma creation	47	11	0.374
Length of operation (min)	177 ± 58	185 ± 76	0.749
Blood loss (g)	222 (0–1372)	326 (0–1000)	0.492

Data are presented as mean ± standard deviation, median (range), or n

^*^ *p* < 0.05

**Table 5:** Multivariate logistic regression analysis of risk factors for mortality of colorectal perforation in the nondiverticular group

Variables	Odds ratio	95 % Confidence interval	*p* value
Age	1.09	1.013–1.182	0.018^*^
Systolic blood pressure	0.98	0.949–1.001	0.064
Heart rate	1.01	0.977–1.055	0.453
Creatinine	1.18	0.633–2.076	0.559

^*^ *p* < 0.05

## Discussion

Despite progress in postoperative management, the prognosis of colorectal perforation remains quite poor. Fecal panperitonitis easily causes septic shock, disseminated intravascular coagulation, and multiple organ failure. Prediction of mortality using routinely and easily available preoperative parameters is important to provide adequate information about the likelihood of postoperative death to patients and their families and prepare for intensive postoperative management in case of the need for rescue.

Our study indicates that higher age and a lower preoperative systolic blood pressure are independent risk factors for mortality in patients with colorectal perforation. Like our study, Kriwanek et al. [7] and Alvarez et al. [2] also indicated that higher age was significantly associated with mortality in patients with colorectal perforation. Our study also indicates that colorectal perforation caused by a nondiverticular etiology is associated with higher mortality than diverticular perforation. Sartelli et al. [1] also indicated in their multicenter trial that nondiverticular perforation was significantly associated with mortality among abdominal infectious diseases. Although these data do not delineate the pathophysiology of this relationship, one could theorize that in patients with diverticular perforation, the size of the perforation is generally smaller than that of perforations of other causes, and this can interrupt the spreading of feces to the peritoneal space, leading to a better prognosis. In this retrospective study, we were not able to obtain adequately detailed information about the size and shape of the perforations and were thus unable to confirm this hypothesis. Further investigations are necessary in this respect.

However, the usefulness of a number of risk score systems has been reported in this field. Sugimoto et al. [10] and Horiuchi et al. [5] reported that a higher Acute Physiology and Chronic Health Evaluation II (APACHE II) score was significantly associated with mortality in patients with colorectal perforation. The APACHE II scoring system was developed by Knaus et al. [12] in 1985 and comprises 12 parameters, including blood pressure, body temperature, respiratory rate, and several laboratory data. The severity of each parameter is classified into nine stages from −4 to +4. We considered that the calculation of this score was too complicated for colorectal perforation in the emergency setting. Bielecki et al. [3] and Kriwanek et al. [7] indicated the usefulness of the Mannheim peritonitis index (MPI) for prediction of mortality in patients with colorectal perforation. The MPI was developed by Linder et al. [13] in 1987 and has since been used to predict mortality associated with peritonitis. One parameter of this scoring system, namely the preoperative duration of peritonitis, is sometimes difficult to determine, especially in patients with impaired consciousness. Whether peritonitis is diffuse or focal may also be difficult to ascertain. We considered the fact that both scoring systems were developed approximately 30 years ago and that recent progress has been made in intensive postoperative management regimens, including the use of several medications such as antibiotics. For this reason, we did not use these scoring systems. We consider that the optimal parameters should be able to be easily determined; therefore, in our analysis, we selected parameters that can be determined easily and routinely in the emergent setting. Ishizuka et al. [14] indicated that the Physiologic and Operative Severity Score for the enUmeration of Mortality (POSSUM) was a sensitive system for predicting mortality associated with colorectal perforation. This scoring system was presented in the early 1990s by Copeland et al. [15, 16], and it is reportedly a useful predictor of postoperative mortality. It includes all parameters that were considered to be associated with mortality in the present study: age, preoperative blood pressure, and preoperative serum creatinine level. However, we were unable to retrospectively analyze one parameter of this scoring system, namely respiratory history; we were therefore unable to determine its usefulness.

The serum procalcitonin level is reportedly a useful marker for the diagnosis and severity of peritonitis according to several studies [17–20]. Pupelis et al. [19] analyzed 222 patients and found that higher procalcitonin levels were associated with an increased risk for septic shock. Additionally, Shimazaki et al. [8] indicated that the serum lactate level could be a predictive marker for mortality in patients with colorectal perforation. They indicated in their retrospective analysis that the postoperative serum lactate level was an independent risk factor for mortality in these patients. These parameters are not routinely determined in our institution. The usefulness of these factors should be analyzed in further investigations.

There were several limitations in this study. First, the operative and postoperative management was performed by different doctors and was thus inconsistent in quality. Additionally, this study was conducted at a single center, and the number of patients was small. A large-scale multicenter study should be performed to confirm our findings.

## Conclusions

Patient age and preoperative blood pressure are useful for prediction of postoperative mortality in patients with colorectal perforation. For older patients and patients with lower blood pressure, adequate information about this higher mortality rate should be provided to the patients and their family members, and postoperative management should be carefully performed.
